# A modular RNA delivery system comprising spherical nucleic acids built on endosome-escaping polymeric nanoparticles†

**DOI:** 10.1039/d2na00846g

**Published:** 2023-05-10

**Authors:** Antonio Garcia-Guerra, Ruth Ellerington, Jens Gaitzsch, Jonathan Bath, Mahnseok Kye, Miguel A. Varela, Giuseppe Battaglia, Matthew J. A. Wood, Raquel Manzano, Carlo Rinaldi, Andrew J. Turberfield

**Affiliations:** a Department of Physics, Clarendon Laboratory, University of Oxford Parks Road Oxford OX1 3PU UK; b Department of Paediatrics, University of Oxford Le Gros Clark Building, South Parks Road Oxford OX1 3QX UK; c Kavli Institute for Nanoscience Discovery, University of Oxford Dorothy Crowfoot Hodgkin Building, South Parks Road Oxford OX1 3QU UK andrew.turberfield@physics.ox.ac.uk +44-1865-272359; d Institute of Developmental and Regenerative Medicine (IDRM) IMS-Tetsuya Nakamura Building, Old Road Campus, Roosevelt Dr, Headington Oxford OX3 7TY UK antonio.garciaguerra@paediatrics.ox.ac.uk +44-1865-272166; e Department of Chemistry, University College London London WC1H 0AJ UK; f Leibniz Institute for Polymer Research Dresden Hohe Str. 6 01069 Dresden Germany; g Institute for Bioengineering of Catalonia, Barcelona Institute of Science and Technology Baldiri Reixac, 10-12 08028 Barcelona Spain; h Catalan Institution for Research and Advanced Studies Passeig de Lluís Companys, 23 08010 Barcelona Spain; i Department of Anatomy, Embryology and Animal Genetics, University of Zaragoza Zaragoza 50013 Spain

## Abstract

Nucleic acid therapeutics require delivery systems to reach their targets. Key challenges to be overcome include avoidance of accumulation in cells of the mononuclear phagocyte system and escape from the endosomal pathway. Spherical nucleic acids (SNAs), in which a gold nanoparticle supports a corona of oligonucleotides, are promising carriers for nucleic acids with valuable properties including nuclease resistance, sequence-specific loading and control of receptor-mediated endocytosis. However, SNAs accumulate in the endosomal pathway and are thus vulnerable to lysosomal degradation or recycling exocytosis. Here, an alternative SNA core based on diblock copolymer PMPC_25_–PDPA_72_ is investigated. This pH-sensitive polymer self-assembles into vesicles with an intrinsic ability to escape endosomes *via* osmotic shock triggered by acidification-induced disassembly. DNA oligos conjugated to PMPC_25_–PDPA_72_ molecules form vesicles, or polymersomes, with DNA coronae on luminal and external surfaces. Nucleic acid cargoes or nucleic acid-tagged targeting moieties can be attached by hybridization to the coronal DNA. These polymeric SNAs are used to deliver siRNA duplexes against *C9orf72*, a genetic target with therapeutic potential for amyotrophic lateral sclerosis, to motor neuron-like cells. By attaching a neuron-specific targeting peptide to the PSNA corona, effective knock-down is achieved at doses of 2 particles per cell.

## Abstract 2

Polymeric spherical nucleic acids comprise pH-sensitive, polymer-conjugated oligonucleotides that self-assemble into nanoparticles with the ability to escape endosomes, overcoming a major obstacle in nucleic acid delivery.

## Graphical abstract

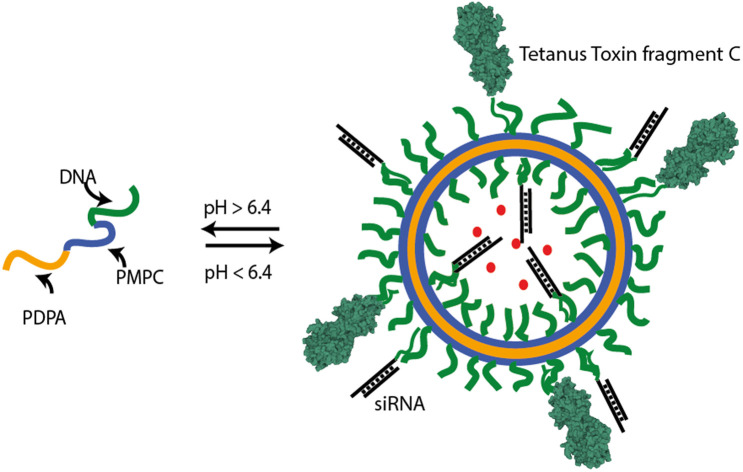

## Introduction

Nucleic acids have great potential to treat disease, for example, through replacement or repair of a defective gene or selective gene silencing.^1^ However, nucleic acids are rapidly degraded by nucleases found in biological fluids^2^ and do not cross the cell membrane directly. Nucleic acid therapeutics are therefore chemically modified or formulated with delivery systems to enable them to reach relevant organs and there to access cytoplasmic targets.^3,4^ Delivery systems are limited by tissue tropism,^5^ immunogenicity^6,7^ and endosomal accumulation.^8^ When using nanoparticle- and virus-based systems it is difficult to avoid filtering organs such as the liver:^5,9,10^ large doses are often required to reach therapeutic levels in a target tissue, increasing cost and toxicity.^11^ Nanoparticles that are taken up *via* the endosomal pathway must escape to avoid exocytosis through recycling or degradation within lysosomal compartments.^12,13^ Escape from endosomes to the cytosol may be accomplished using molecules that disrupt or fuse with the endosomal membrane.^8^

Spherical nucleic acids (SNAs) are dense, oriented oligonucleotide brushes arranged in a spherical shell on a nanoparticle template, normally gold.^14,15^ The biological activity of an SNA arises from its nucleic acid shell: SNAs from which the core has been dissolved retain their characteristic properties.^16,17^ Polyvalent SNAs bind nucleic acid targets with high affinity^18^ and the dense oligonucleotide shell protects against nuclease activity.^19^ Cell entry is mediated by class A scavenger receptors.^20^ SNAs can mediate gene silencing *in vitro* and *in vivo*^20–22^ but accumulation in the endosomal pathway^23^ limits their potency. SNAs can be complexed with cationic polymers to increase endosomal escape^24^ but at the expense of reduced control over key physical properties of the nanoparticle (*e.g.* charge, size, hydrophobicity, *etc.*) that strongly affect biodistribution.^25–27^ Alternative SNA cores can provide new physical properties.^28–30^ Hence, optimization of the carrier nanoparticle is essential for the development of better SNA delivery systems.

**Fig. 1:**
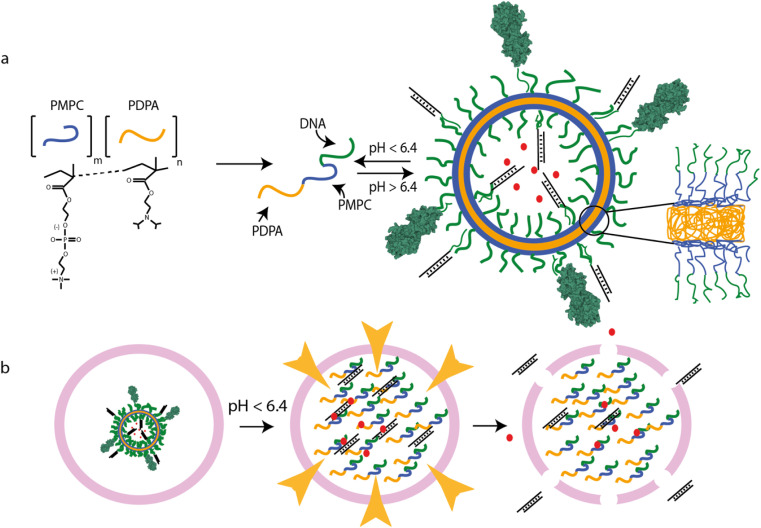
PSNA structure and function. (a) pH-dependent self-assembly of polymersomes from PMPC_25_–PDPA_72_ diblock copolymers. PMPC is zwitterionic; PDPA is pH-sensitive, with tertiary amines that protonate at low pH (p*K*_a_ = 6.4). At higher pH, the amphiphilic polymer forms polymersomes with bilayer membranes. Hydrophobic PDPA blocks are buried within the membrane; hydrophilic PMPC blocks are exposed to the lumen and extravesicular medium. DNA oligonucleotides conjugated to the PMPC terminus form internal and external coronae which can be used for sequence-specific loading of DNA- or RNA-tagged molecules, for instance siRNA duplexes or targeting moieties such as tetanus toxin fragment C (structure 1DFQ).^52,53,61^ Unbound cargoes can also be encapsulated in the lumen. (b) PMPC_25_–PDPA_72_ vesicles escape the endosomal pathway *via* pH-driven osmotic shock. When endosomal acidification reaches a pH below the p*K*_a_ of PMPC_25_–PDPA_72_ the vesicle disassembles, solubilizing thousands of polymer molecules within the endosome. The resulting osmotic shock can transiently disrupt the endosomal membrane, allowing therapeutic cargoes to access the cytosol.

PMPC–PDPA (poly(2-(methacryloyloxy)ethyl phosphorylcholine)–poly(2-(diisopropylamine)ethyl methacrylate)) is a diblock copolymer comprising zwitterionic PMPC, which is hydrophilic, and ionizable PDPA whose solubility in water is pH-dependent.^31^ The polymer self-assembles reversibly to form vesicles: assembly depends on pH and temperature.^32^ At high pH (significantly above the p*K*_a_ of the PDPA moiety) the PDPA is uncharged and hydrophobic: the diblock copolymer is thus amphiphilic and assembles in aqueous solution to form stable vesicles. The PDPA is protonated and hydrophilic at lower pH so vesicles do not form. This pH-dependent transition can be used to control the assembly of the vesicles and underlies their endosome-escaping abilities^33^ (Fig. 1a). When maturation-associated acidification within endosomal compartments triggers vesicle disassembly, each vesicle releases thousands^31,34^ of polymer molecules. The resultant transient increase in osmotic pressure disrupts the endosome, forming pores through which the cargo can enter the cytosol (Fig. 1b). The observed p*K*_a_ of PMPC_25_–PDPA_72_ (pH 6.4)^31^ is similar to those of the most efficient fusogenic ionizable cationic lipids used for siRNA delivery.^35^ PMPC_25_–PDPA_72_ polymersomes are effective in intracellular delivery of paclitaxel,^36^ antibodies^37^ and plasmid DNA;^38,39^ ligand-functionalized polymersomes can cross the blood–brain barrier.^37^

Here, we explore the use of self-assembled PMPC_25_–PDPA_72_ vesicles as nanoparticle templates on which to build SNAs. The resulting polymeric spherical nucleic acids (PSNAs) are designed to combine the endosomal escape functionality of PMPC_25_–PDPA_72_ polymersomes with the protective properties and ease of loading and particle decoration provided by the DNA corona of the SNA. We explore the therapeutic potential of PSNAs by delivering siRNA duplexes against *C9orf72*, a target for genetic treatment of the neurodegenerative disease amyotrophic lateral sclerosis (ALS), to motor-neuron-like cells.

## Experimental

### Polymer–DNA conjugation

Preparation of DNA-functionalized PMPC_25_–PDPA_72_ starts with the pre-assembly of micelles from the thiol-modified copolymer (ESI†) in reduced form: PDPA_72_–PMPC_25_-SS-PMPC_25_–PDPA_72_. A disulphide PMPC_25_–PDPA_72_ stock solution is made by dissolving the polymer in phosphate-buffered saline (PBS, Gibco) adjusted to pH 2 with hydrochloric acid (1 N). Aliquots containing 2 mg (44 nmol) polymer are heated to 50 °C in a water bath. 0.5 M sodium hydroxide is added to adjust to approximately pH 8 and the aliquots vortexed vigorously. Each aliquot is topped up to 800 μL with pH 7.4 PBS and maleimide–DNA in 5× molar excess over thiol–PMPC_25_–PDPA_72_ (see ESI† for oligonucleotide sequences and functionalization). The solution is adjusted to pH 7.4 and purged for 30 minutes with argon. 2.5 mM tris(2-carboxyethyl)phosphine (TCEP, Thermo Scientific) in 200 μL PBS (pH 7.4) is added to cleave the disulphide bond, starting the thiol–maleimide conjugation. The reaction is incubated at room temperature while stirring for 48–72 h in an argon atmosphere.

After completion of the reaction the solution is ultrafiltered (Amicon Ultra 0.5 mL, 30 kDa cut-off). Filtration is repeated 8 times, topped up every time with water pH 8 to ensure removal of excess DNA and complete buffer exchange for lyophilization. 400 μg of PSNA solution is stored for HPLC analysis; the rest is frozen and lyophilized for storage.

### Tetanus toxin fragment C: conjugation to DNA

100 pmol tetanus toxin fragment C (TTC) is mixed with 4 nmol maleimide–DNA (complementary to the DNA in the PSNA corona) in 30 μL PBS pH 7.4 containing 500 μM TCEP. The reaction is left overnight at 4 °C in an orbital shaker. Conjugates are analyzed by polyacrylamide gel electrophoresis using a Bolt gel (Invitrogen) run at 200 V for 32 minutes in MES buffer (Invitrogen) and stained with Sypro Ruby (Invitrogen) following manufacturer's instructions (ESI Fig. 1a†). TTC–DNA conjugates are purified using size-exclusion liquid chromatography (SE-LC) with a 10/300 column pre-packed with Superdex Peptide (GE) (ESI Fig. 1b†). Conjugates are injected using a 0.5 mL loop. The column is eluted with 1.5 column volumes of PBS at 0.5 mL min^−1^. Conjugates elute between minutes 16 and 20. Purified conjugates are concentrated 3× using an Amicon Ultra 0.5 mL 30 kDa centrifugal filter (Millipore).

### PSNA and polymersome assembly and purification

DNA-functionalized and unmodified PMPC_25_–PDPA_72_ molecules (molar ratio 1 : 1, final total polymer concentration 2 mg mL^−1^) are dissolved in 800 μL PBS adjusted to pH 2 with HCl. The solution is introduced in a 5 mL balloon flask with a mechanical stirrer, a pH probe and a needle connected to a syringe pump (KD Scientific model 210). While stirring, PBS supplemented with NaOH to a concentration of 0.1 M is added at a rate of 2 μL min^−1^ until pH 5 is achieved. Any cargo is added (rhodamine at a final concentration of 1 mg mL^−1^ or siRNA at a 10% molar ratio to polymer molecules) and the pump rate is decreased to 1 μL min^−1^ until pH 7.4 is reached, at which point the PSNAs are formed.

PSNAs are ultracentrifuged at 20,000*g* for 10 minutes to remove aggregates then further purified by SE-LC using a 10/300 ÄKTA column packed with Sepharose 4 Fast Flow resin (GE Healthcare) and connected to an ÄKTA Pure Chromatography System. PSNA nanoparticles are injected using a 1 mL loop. The column is eluted with 1.5 column volumes of PBS at 0.5 mL min^−1^. Absorption at 280 nm (indicating the presence of DNA whose maximum absorption is at 260 nm) is monitored. PSNA nanoparticles elute between minutes 6 and 12; excess cargo and polymer elute later. When adding TTC, the DNA-conjugated peptide is incubated with the PSNA for 1 h immediately after removing the aggregates and before SE-LC purification.

### Statistical analysis

The statistical significance of comparison between a pair of conditions was calculated using an unpaired, two-tailed *t*-test in GraphPad Prism 9. For groups of three or more, one-way ANOVA with Dunnett's multiple comparison correction was used.

## Results & discussion

**Fig. 2:**
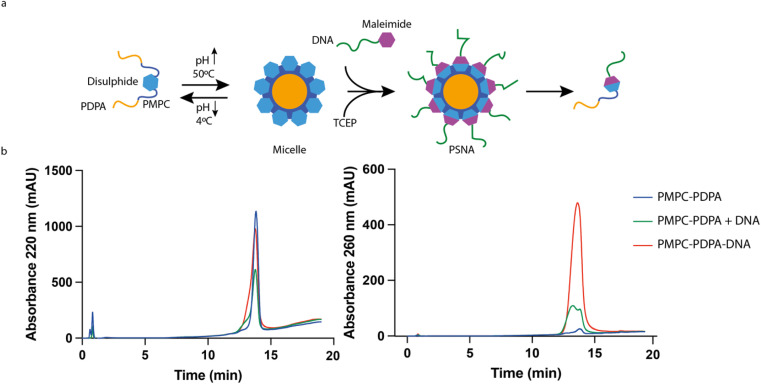
DNA–polymer conjugation. (a) Micelle reaction scheme. To circumvent strong electrostatic interactions between DNA and the ionized PDPA block the conjugation reaction is carried out at 50 °C and basic pH, under which conditions PDPA is hydrophobic and the polymer forms micelles. Once the reaction has finished, the micelles are disassembled by reducing the pH. (b) HPLC analysis of purified reaction products with controls (polymer only, unfunctionalized polymer + DNA). Both polymer and DNA absorb at 220 nm; only DNA absorbs significantly at 260 nm. The DNA-functionalized polymer, identified by its absorption at 260 nm, elutes at minute 13.8, close to the elution time of unfunctionalized PMPC_25_–PDPA_72_.

The molecular building blocks of PSNAs are produced by covalently linking DNA oligonucleotides to PMPC_25_–PDPA_72_ (Fig. 1a) adapting a previously reported ligation protocol:^40^ amino-modified 25-mer oligonucleotides were reacted with hetero-bifunctional crosslinker *N*-succinimidyl 6-maleimidohexanoate (EMCS) (ESI Fig. 2†) and the resulting maleimide-functionalized DNA coupled to the thiolated unimer HS–PMPC_25_–PDPA_72_. The polyanionic PDPA moiety of soluble PMPC_25_–PDPA_72_ binds strongly to polycationic DNA^38^ (ESI Fig. 3†), impeding the coupling reaction. To avoid this, we perform the reaction with pre-assembled PMPC_25_–PDPA_72_ micelles, formed at 50 °C ^32^ (ESI Fig. 4†), such that the PDPA block is sequestered within the micelle core (Fig. 2a). Fig. 2b shows HPLC analysis of reaction products (ESI Fig. 5†) after ultrafiltration to remove unreacted DNA.

**Fig. 3:**
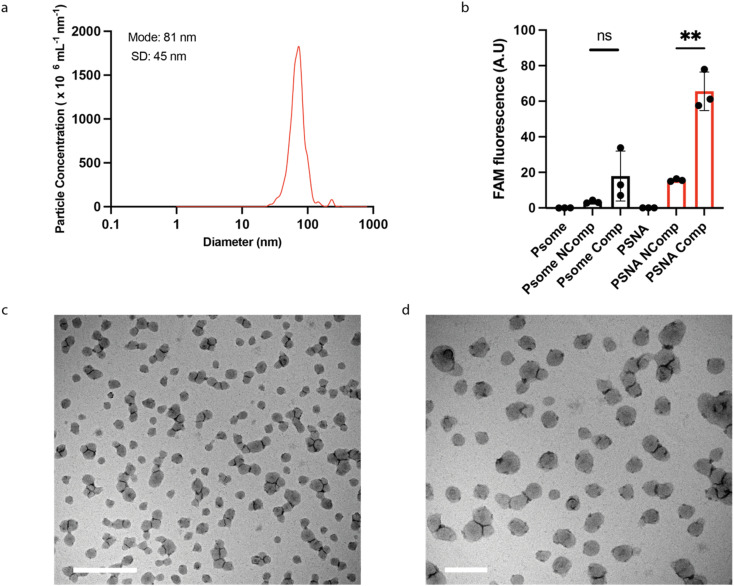
PSNA characterization. (a) Particle size distributions of PSNAs measured by Nanoparticle Tracking Analysis. (b) Sequence-specific labelling of external corona by hybridization to a complementary oligonucleotide. PSNAs and non-DNA-functionalized PMPC_25_–PDPA_72_ polymersome controls were challenged with FAM-labelled oligonucleotides complementary (Comp) and non-complementary (Ncomp) to the oligonucleotides in the PSNA corona. Unbound DNA was removed by ultrafiltration. Highest loading is achieved by the PSNA sample for which the labelled oligo is complementary to the corona. Data are presented as mean and SD (*n* = 3). Samples were compared using unpaired two-tailed *t*-tests **p < 0.01. (c) and (d) Representative transmission electron micrographs of PSNAs positively stained with 0.75% phosphotungstic acid solution adjusted to pH 7.4 to prevent PSNA disassembly. Left, magnified view of the grid. Scale bars: 1 μm (c); 200 nm (d).

PSNAs were assembled by using a syringe pump to increase slowly the pH of a solution of DNA-functionalized polymer. PSNAs formed at pH 7.4 were purified by size-exclusion liquid chromatography (SE-LC). No vesicles were observed using 100% DNA-functionalized polymer (ESI Fig. 6a†). We hypothesize that electrostatic and steric interactions of the conjugated DNA moieties change the effective shape and solubility of the polymer and thus its ability to self-assemble into vesicles.^32^ A 1 : 1 (by mass) mixture of DNA-functionalized and unmodified polymer does reproducibly form vesicles (ESI Fig. 6b†) with diameters of 70–90 nm as determined by Nanoparticle Tracking Analysis^41^ (Fig. 3a). Transmission electron microscopy (TEM) micrographs (Fig. 3c, d and ESI Fig. 7†) show morphology similar to that previously reported.^32,39^

To test whether the conjugated DNA is available for hybridization with complementary oligonucleotides, we challenged assembled PSNAs with fluorescently labelled complementary and non-complementary oligos (Fig. 3b). Only complementary DNA bound significantly to the PSNA, consistent with sequence-specific hybridization to the DNA corona.

**Fig. 4:**
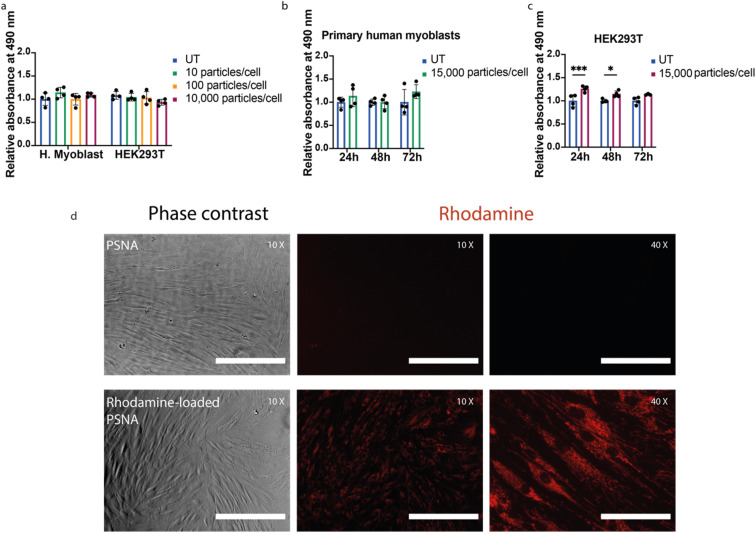
Cell proliferation assays and cargo delivery. (a) Cell viability as a function of PSNA dose for primary human myoblasts and HEK293T cells. After incubation for 24 h with 10, 100 and 10,000 PSNAs per cell, cell proliferation was measured using a CellTiter (MTS) assay. Data presented is mean and SD (*n* = 4); samples were compared using a two-way ANOVA with Bonferroni's multiple comparison test. (b) Time-course cell proliferation assay using primary human myoblasts. Proliferation of primary human myoblasts was measured after 24, 48 and 72 h incubation with 15,000 PSNAs per cell and compared to untreated control. Data are presented as mean and SD (*n* = 4); samples were compared using a two-way ANOVA with Bonferroni's multiple comparison test. (c) Time-course cell proliferation assay using HEK293T cells. Conditions as (b). *p < 0.05, ***p < 0.001. (d) Delivery of Rhodamine 6G to human primary fibroblasts. 50,000 primary human fibroblasts per well were treated 24 h before imaging with 10^10^ PSNAs loaded with rhodamine during PSNA vesicle formation. Treated samples were compared with cells treated with an empty PSNA control. Images are representative. Scale bars 400 μm (10× images), 100 μm (40× images).

PMPC_25_–PDPA_72_ is known to be well tolerated *in vitro*^33^ and *in vivo*.^37^ To test whether a DNA corona increases toxicity, we exposed primary human myoblasts and HEK293T cells to PSNAs. We observed no reduction in cell proliferation compared to untreated controls (Fig. 4a–c).

To assess the ability of PSNAs to deliver cargoes to the cytoplasm, we loaded the PSNA lumen with rhodamine. Primary human fibroblasts treated with rhodamine-loaded PSNAs showed high levels of particle uptake (Fig. 4d): rhodamine fluorescence was distributed across the cytosol.^33^ We also used PSNAs to deliver a DNA oligo labelled with fluorophore Cy5.5 to primary human myoblasts (ESI Fig. 8†). After overnight incubation some colocalization of Cy5.5 fluorescence with acidified compartments (lysosomes), characteristic of non-productive delivery,^42^ was observed, but the majority of the fluorescence was localised elsewhere in extended structures. Both results are consistent with endosomal escape of the cargo.^33,42^

We used siRNA^43^ targeting the gene *C9orf72* to explore the use of PSNAs to deliver therapeutically relevant cargoes and the potential of modifying the PSNA surface with targeting molecules for cell-type-specific delivery. We chose siRNA as our model nucleic acid therapeutic for its potential in gene therapy,^3^ its track record in the clinic with drugs such as patisiran^44^ and givosiran,^45^ and because there is a pressing need for effective delivery systems that enable tissue-specific cytosolic delivery. *C9orf72* is a therapeutic target for the treatment of amyotrophic lateral sclerosis,^46–49^ a devastating and currently incurable adult-onset neurodegenerative disease characterized by the progressive degeneration of motor neurons in the brain and spinal cord. The most common implicated mutation is an expansion of a hexanucleotide repeat (GGGGCC) in *C9orf72*.^49^ Knock-down of *C9orf72* using anti-sense oligonucleotides ameliorates the phenotype in patient-derived iPS motor neurons.^50,51^ To test the potential of our PSNAs in therapy, we developed a *C9orf72*-specific siRNA molecule able to hybridize with the DNA corona.

We tested *in vitro* several designs for a siRNA duplex modified by addition of a sequence complementary to the DNA strands of the PSNA corona as a 3′ extension to the sense strand (ESI table†). siRNA potency is slightly reduced when this extension is hybridized with a complementary DNA strand (ESI Fig. 9a†). Attempts to restore full activity by using a polyA spacer (A_5_ or A_10_) to separate the siRNA duplex from the DNA–RNA heteroduplex did not improve the knock-down efficiency (ESI Fig. 9b†). We therefore continued with a *C9orf72* siRNA lacking a spacer.

**Fig. 5:**
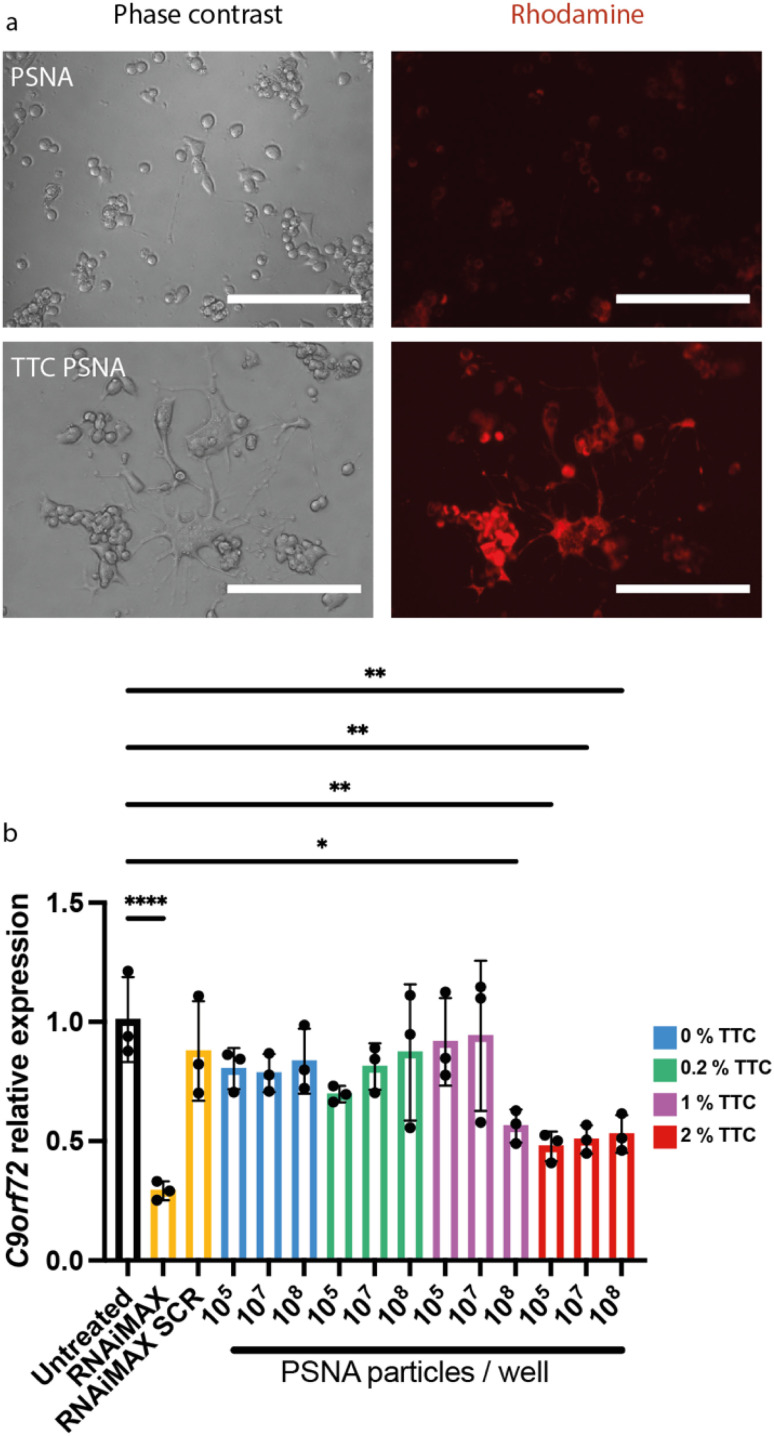
PSNAs loaded with targeting peptide tetanus toxin fragment C deliver a small molecule (Rhodamine 6G) and siRNA to neuronal cells. (a) Delivery of encapsulated Rhodamine 6G. NSC34 cells were incubated overnight with rhodamine-loaded PSNAs (2 × 10^10^ per well) with and without targeting peptide TTC (2% mol fraction w.r.t. polymer). Scale bars 200 μm. (b) Knockdown of *C9orf72* in NSC34 cells by siRNA tethered to the PSNA corona. PSNAs loaded with siRNA (10% molar ratio to polymer molecules) were used to evaluate the effects of particle dose and of surface density of targeting ligand TTC on gene expression. 2% TTC loading dramatically increases delivery efficiency: the dose at which maximum knock-down is accomplished is reduced by three orders of magnitude. All conditions used 50,000 cells per well, harvested 48 h after treatment. Controls: siRNA and a scramble control (SCR) delivered using cationic lipid transfection agent Lipofectamine™ RNAiMAX at a final concentration of 50 nM. Data presented is mean and SD (*n* = 3). Samples were compared using a one-way ANOVA with Dunnett's multiple comparison test *p < 0.05, **p < 0.01, ****p < 0.0001.

To enhance PSNA delivery and promote specific neuronal uptake we added a targeting peptide derived from *Clostridium tetani* toxin. The peptide is bound to the corona through a conjugated DNA oligonucleotide. *Clostridium tetani* toxin comprises a light chain, containing catalytic and toxic domains, and a heavy chain containing translocation and receptor-binding domains. The C-terminal fragment of the heavy chain (TTC) retains the ability to bind neuronal membranes: binding is followed by internalization and retrograde axonal transport with final accumulation in the motor neuron.^52,53^ We reacted TTC (which contains cysteine residues) with maleimide–DNA to form conjugates that hybridize with the PSNA corona (ESI Fig. 1†). To confirm that TTC-decorated PSNAs can deliver cargoes to the cytosol of motor neurons, we used them to deliver rhodamine to motor-neuron-like NSC34 cells: there is a large increase in rhodamine internalization when PSNAs are decorated with TTC (Fig. 5a).

To evaluate the performance of our PSNAs in delivering siRNA, we quantified gene knock-down in NSC34 cells treated with PSNAs carrying a fixed quantity of siRNA against *C9orf72*. The modified siRNA was added during vesicle assembly and is expected to hybridize to the DNA coronae on both interior and exterior surfaces. Based on the stoichiometry of the PSNA assembly mixture and an estimate of 1000 polymer molecules per particle,^31,34^ each particle is expected to carry approximately 50 siRNA duplexes. We titrated the surface density of TTC on the vesicles and the PSNA dose (Fig. 5b). Results showed that TTC is required for gene knock-down: no knock-down is observed in the absence of TTC or at low loadings (0.2% mol fraction w.r.t. polymer). At 1% TTC we observe a dose-dependent response, with statistically significant knock-down only at the highest particle dose tested (2000 particles per cell). A higher density of targeting ligand dramatically increases delivery efficiency. With 2% TTC we observe knock-down of *C9orf72* at all doses tested. Maximum knock-down is achieved with a RNAi dose equivalent to 2 PSNAs per cell, three orders of magnitude lower than with 1% TTC and three orders of magnitude less than the Lipofectamine™ RNAiMAX positive control.

## Conclusion

Polymersomes formed from the pH-responsive diblock copolymer PMPC_25_–PDPA_72_ can be used to template spherical nucleic acids. The resulting composite nanoparticles, polymeric spherical nucleic acids, share the characteristics of more conventional SNAs: hybridization of complementary oligonucleotides to the DNA corona can be used to load nucleic acids or other nucleic acid-tagged cargoes, for example, siRNA duplexes or targeting ligands. The most significant additional property conferred by the polymeric core is an intrinsic ability to escape the endosomal pathway and thus deliver cargoes to the cytosol.

We have demonstrated the ability of PSNAs to deliver small hydrophilic molecules and siRNA to motor-neuron-like cells *in vitro*, confirming the therapeutic potential of the technology. PSNAs produce knock-down equivalent to state-of-the-art transfection reagent Lipofectamine™ RNAiMAX with doses three orders of magnitude lower. This indication of excellent cytosolic delivery efficiency is promising: an important factor limiting the adoption of nucleic acid therapies is the need for high doses to compensate for off-target accumulation, with consequent high cost and increased toxicity.

These DNA–polymer conjugates could be exploited for other important biomedical applications, including the delivery of larger therapeutic nucleic acids such as mRNA or plasmid DNA.^38,39^ This could give rise to combination therapies where PSNAs simultaneously deliver siRNA against a mutated gene and a corrected gene copy. Such a therapy for genetic diseases with a mixed toxic gain- and loss-of-function mechanism, such as ALS,^46–49^ could overcome potential long-term loss-of-function toxicity caused by knockdown of the mutated gene^54^ without functional replacement. The DNA corona inside PSNAs could be used to load DNA nanostructures^55–58^ which have the potential to probe and manipulate cellular behaviour but which are unstable in biological fluids.^59^ The attached oligonucleotides could also be used as molecular barcodes to screen polymer libraries for their potential in therapeutic delivery.^60^

## Author contributions

Conceptualization: AGG (lead), AJT, RM, GB. Data curation: AGG (lead), AJT, CR, RE. Formal analysis: AGG (lead), AJT, CR, RE. Funding acquisition: AJT, RM (leads), AGG, CR, MAV, MJAW. Investigation: AGG (lead), AJT, RM, CR, RE. Methodology: AGG (lead), AJT, JG, MK, JB, GB. Project admin.: AGG (lead), AJT. Resources: AJT (lead), AGG, RM, CR, JG, GB, MJAW. Supervision: AGG (lead), AJT, RM, CR, MJAW. Validation: AGG (lead), AJT, RM, CR. Visualization: AGG (lead), AJT. Writing – original draft: AGG. Writing – review and editing: AGG, AJT (leads) with all authors contributing.

## Conflicts of interest

The authors declare no conflicts of interest.

## Supplementary Material

NA-005-D2NA00846G-s001
