# Supplementary material for: A modular RNA delivery system comprising spherical nucleic acids built on endosome-escaping polymeric nanoparticles
Source: Nanoscale Adv. 2023 May 10;5(11):2941–9. doi: 10.1039/d2na00846g (PMC10228346; doi:10.1039/d2na00846g)
Supplement: NA-005-D2NA00846G-s001 [file NA-005-D2NA00846G-s001.pdf]

Electronic Supplementary Material (ESI) for Nanoscale Advances.
This journal is © The Royal Society of Chemistry 2023

Electronic Supplementary Information

A modular RNA delivery system comprising Spherical Nucleic Acids built on endosome-escaping polymeric nanoparticles

Antonio Garcia-Guerra[a,b, c, d]*, Ruth Ellerington[b,d], Jens Gaitzsch[e,f], Jonathan Bath[a,c], Mahnseok Kye[b,d], Miguel A. Varela[b, d], Giuseppe Battaglia[e,g,h], Matthew J. A. Wood[b,d], Raquel Manzano[b,i†], Carlo Rinaldi[b,d], Andrew J. Turberfield[a,c*†].

a. Department of Physics, University of Oxford, Clarendon Laboratory, Parks Road, Oxford OX1 3PU, United Kingdom.
b. Department of Paediatrics, University of Oxford, Le Gros Clark Building, South Parks Road, Oxford, OX1 3QX, United Kingdom.
c. Kavli Institute for Nanoscience Discovery, University of Oxford, Dorothy Crowfoot Hodgkin Building, South Parks Road, Oxford, OX1 3QU, United Kingdom.
d. Institute of Developmental and Regenerative Medicine (IDRM), IMS-Tetsuya Nakamura Building, Old Road Campus, Roosevelt Dr, Headington, Oxford, OX3 TY, United Kingdom.
e. Department of Chemistry, University College London, London, WC1H 0AJ, United Kingdom.
f. Leibniz Institute for Polymer Research Dresden, Hohe Str. 6, 01069 Dresden, Germany
g. Institute for Bioengineering of Catalonia, Barcelona Institute of Science and Technology, Baldiri Reixac, 10-12, 08028 Barcelona, Spain
h. Catalan Institution for Research and Advanced Studies, Passeig de Lluís Companys, 23, 08010 Barcelona, Spain
i. Department of Anatomy, Embryology and Animal Genetics, University of Zaragoza, Zaragoza, 50013, Spain.

Supplementary methods

Reagents

All reagents were bought from Sigma unless otherwise stated.

Thiolated polymer synthesis

2-(Methacryloyloxy)ethyl phosphorylcholine monomer (MPC, 99.9% purity) was donated by Biocompatibles U.K. Ltd. Diisopropylaminoethyl methacrylate (DPA), alpha-bromoisobutyryl bromide, bis[2-(2'-bromoisobutyryloxy)ethyl]disulfide] (98 %), silica gel 0.2 - 0.5mm 30-70 mesh chromatograph, copper(I) bromide (Cu(I)Br, 99.999%), trifluoroacetic acid, 2,2'-bipyridine (bpy, 99%), chloroform, poly(ethylene glycol) azide (M_n = 1500), ethyl-4-bromobutyrate (95 %), and n-hexane (≥ 97 %) were purchased from Sigma Aldrich UK. Methananol (Normapur) was purchased from Merck KG (Darmstadt, Germany). All of the above were used as received. Semi-permeable cellulose dialysis tubing (Spectra/Por 6 MWCO 1,000) was purchased from Spectrum Labs (Breda, Netherlands).

Thiol-PMPC-PDPA copolymers were synthesized using standard literature procedures for ATRP described previously[40].Briefly, 0.05 mmol of initiator bis[2-(2'-bromoisobutyryloxy)ethyl]disulfide were dissolved in 1mL EtOH in a round-bottom flask and 50 eq. of MPC were added (740 mg, 2.50 mmol, 50 eq.). The mixture was stirred to be homogeneous and purged with nitrogen for 30 minutes at 30 °C. Then, a mixture of bpy (32 mg, 0.20 mmol, 4 eq.) and Cu(I)Br (15 mg, 0.10 mmol, 2 eq.) was added under a constant nitrogen flow. The mixture was stirred for 60 minutes to yield a highly viscous brown substance and sampled for NMR to estimate conversion (gave full conversion, no monomer peaks present). Meanwhile, a solution of DPA (1400 mg, 6.5 mmol, 170 eq.) in 2 mL ethanol was prepared and purged with nitrogen for 60 minutes in a separate flask. Then, the DPA solution was added to the polymerisation mixture, the reaction mixture was purged for another 10 minutes and then left overnight at 30°C. After 18 h, ^{1}H NMR analysis confirmed that the conversion was > 99 % and the reaction was opened to the atmosphere and diluted with ethanol. The solution gradually turned green, indicating oxidation of the copper-based catalyst system. The green solution was passed through silica using ethanol and evaporated partially to give an opaque solution. The solution was then dialyzed (MWCO 1 kDa) against Chloroform/Methanol (vol/vol, 3:1) (2 times), methanol (2 times) and water (2 times) for 8-14 hours each dialysis cycle. The polymer was then freeze-dried under vacuum.

PDPA-PMPC-SS-PMPC-PDPA characteristics: NMR (ppm, ^{1}H, 600 MHz, all broad): 0.85 (CH_3, PMPC+PDPA), 0.98 (CH_3, PDPA)), 1.78 (CH_2, PMPC+PDPA)), 2.61 (CH, PDPA)), 2.95 (CH_2, PDPA), 3.24 (CH_3, PMPC), 3.68 (CH_2, PMPC), 3.81 (CH_2, PDPA), 3.99 (CH_2, PMPC), 4.13 (CH_2, PMPC), 4.23 (CH_2, PMPC). Degrees of Polymerisation: $PDPA_{72}$-$PMPC_{25}$-SS-$PMPC_{25}$-$PDPA_{72}$, M_n = 44.9 kg/mol, Dispersity: 1.24

Maleimide-DNA conjugation

100 nmol lyophilized amine-modified DNA (Integrated DNA Technologies) is resuspended in 10 µL MilliQ water. 184 molar equivalents (18.4 µmol) N-succinimidyl 6-maleimidohexanoate (EMCS, Tokyo Chemical Industries) in dimethylformamide (DMF) and 6.25 µL of N,N-diisopropylethylamine (DIPEA) are mixed, topped up with DMF to a final

volume of 77.75 µL, left rocking for 5 minutes at room temperature, then added dropwise to the DNA in water. The reaction is incubated in a vortex mixer (1400 rpm) for 1 h at room temperature. Maleimide-DNA conjugates are ethanol-precipitated and purified by HPLC using a Waters XBridge Oligonucleotide BEH C18 column (13 nm pore, 2.5 µm particle size, 4.6 mm × 50 mm) in an Agilent Technologies Series 1200 HPLC. Mobile phases: A) 0.1 M triethylammonium acetate (TEAA, Applichem), 5% acetonitrile (HPLC grade, Fisher Scientific); B) 0.1 M TEAA, 70% acetonitrile. Buffers are filtered through a 0.2 µm filter and degassed. Elution program, with flow rate 1 mL/min, interpolates linearly between the following points (minute, % B): (0, 0), (1, 5), (20, 20), (21, 100), (22, 100), (22.10, 0). Fractions containing DNA are identified by their absorbance at 260 nm (Supplementary information figure 2). Maleimide-DNA is dried in a SpeedVac concentrator (DNA Savant 120, Thermo Scientific), resuspended in phosphate-buffered saline (PBS, Gibco) and frozen for later use.

Polymer analytical HPLC and DNA quantification

The HPLC system and column used are described in the Experimental section. The mobile phases were A: Water + 0.05 % trifluoroacetic acid (TFA), B: Methanol + 0.05 % TFA. HPLC flow rate 1 mL/min with elution program (minute, Vol%B): (0, 5), (5, 5), (17, 80), (18.5, 80), (19,5). The column was washed 4 times between samples. Absorbance of the eluant was monitored at 220 nm (polymer+DNA) and 260 nm (DNA) (Figure 2b).

The yield of DNA-polymer conjugate was quantified using the HPLC chromatogram at 260 nm, calibrated using known quantities of DNA (Supplementary information figure 5).

Nucleic acid cargo loading

Nucleic acid cargoes incorporating a domain complementary to the PSNA corona are loaded by addition during PSNA assembly. Random localization (intra and extraluminal) is expected if the cargo is added during nanoparticle self-assembly. Alternatively, exclusive decoration of the PSNA's surface can be accomplished by adding the nucleic acid cargo once assembly is completed. Loading is determined by the number of oligos available for hybridization. We expect an estimate of 1,000 polymer molecules per particle based on the stoichiometry of the PSNA assembly mixture[52,53].

PSNA DNA hybridization assay

2 mg samples of PSNAs and of unfunctionalized polymersomes were prepared and purified by ultracentrifugation as described in the Experimental section. Each was split in two and incubated for 30 minutes at room temperature in PBS buffer with 10 µM FAM-labelled DNA, either non-complementary (Ncomp) or complementary (Comp) to the oligonucleotide forming the PSNA corona. Unbound DNA was removed using an Amicon Ultrafiltration spin filter (0.5 mL, 100 kDa, 4 spins). FAM fluorescence from 1 x 10^{10} particles of each condition was measured in a plate reader (ClarioStar, BMG Labtech) (Figure 3b).

Dynamic Light Scattering

Samples were measured using a Viscotek 802 DLS (Malvern). Software (OmniSIZE 3.0) was set to "auto-attenuation" with a target of 300 k Counts. Samples were measured in acidified (pH 2) and non-pH-adjusted (pH 7.4) PBS. 1-3 groups of 10 runs of 10 seconds were used in order to obtain 5-10 correlation curves that pass quality control which were used to calculate size distributions.

Nanoparticle tracking measurements

Nanoparticle tracking analysis of particle size was performed using a Nanosight NS500 (Malvern, software version 2.3). PSNA nanoparticles were diluted in PBS to a concentration in the range 2-9×10^8 particles per mL. Samples were recorded 5 times for 30 seconds (camera level 14, screen gain 1). Recordings were analysed with screen gain 10 and detection threshold 7.

Cell viability assay

For dose-response experiments (Figure 4a), 20,000 primary human myoblasts and HEK293T were seeded in a 96 well plate and incubated with 10, 100 and 10,000 PSNAs per cell for 24h. Cell proliferation was measured using Promega CellTiter 96® Aqueous One Solution Cell Proliferation Assay (MTS) Kit following the manufacturer's instructions.

For time course experiments (Figure 4b,c), 7,000 primary human myoblasts and HEK293T were seeded in a 96 well plate and incubated with 15,000 PSNAs for 24, 48 and 72h. Cell proliferation was measured using Promega CellTiter 96® Aqueous One Solution Cell Proliferation Assay (MTS) Kit following the manufacturer's instructions.

Cell culture

HEK293T and NSC34 cells were cultured in full Dulbecco's Modified Eagle Medium (DMEM + Glutamax, Gibco) supplemented with 10% Fetal Bovine Serum (Gibco) and 1% Penicillin-Streptomycin. Primary human myoblasts were cultured using Skeletal Muscle Cell Growth medium supplemented with 1% Antibiotic/Antimycotic (sigma) and Supplement Skeletal Muscle Cell Growth Medium (PromoCell).

Rhodamine delivery to NSC34

50,000 human primary fibroblasts were incubated with 1x10^{10} PSNAs, either empty or loaded with rhodamine (Thermo Scientific), as described in the Experimental section. After 24 h the medium was changed and cells imaged.

Rhodamine-loaded PSNAs were prepared with and without addition of TTC ligand (2% mol fraction w.r.t. polymer) after assembly. 2 x 10^{10} particles were added to 50,000 NSC34 cells and incubated overnight. The medium was then changed and cells imaged.

All images were taken in an inverted epifluorescence microscope (Evos Fl, Thermo Scientific).

Confocal imaging of primary human myoblasts.

30,000 primary human myoblasts were seeded in a μ-slide 8 well Ibidi chamber and treated overnight with 2x10^8 PSNAs loaded with 100 Cy5.5 oligos per particle. Cells were labelled

with LysoTracker Green DND-26 (Thermo) and Hoescht 33342 (sigma) following the manufacturer's instructions. Cells were imaged in Hank's Balanced Salt Solution (HBSS) from Gibco, supplemented with 10 mM HEPES and 5% Fetal Bovine Serum (FBS), using a ZEISS 980 IDRM Airyscan 2 with (s/p)FCS.

siRNA delivery to NSC34 cells

1 mg of total polymer, comprising 0.5 mg DNA-$PMPC_{25}$-$PDPA_{72}$ conjugate (11 nmol) and 0.5 mg unmodified $PMPC_{25}$-$PDPA_{72}$ (11 nmol) was assembled into PSNAs in the presence of siRNA (2.2 nmol, i.e. 10% mol fraction w.r.t. total polymer) and purified by SE-LC. Assembled PSNAs were decorated with different densities of TTC (0, 0.2, 1, 2% mol fraction w.r.t. total polymer). 50,000 NSC34 cells were incubated for 48 h with different doses of siRNA-loaded TTC PSNAs. siRNA and siRNA scramble controls were transfected using Lipofectamine™ RNAiMAX (Invitrogen) following the manufacturer's protocol at a final siRNA concentration of 50 nM. After 48 h the cells were prepared for RNA extraction and qPCR.

RNA extraction, cDNA synthesis, and qPCR

RNA extraction was performed with a Rneasy Mini Kit (Qiagen) following the manufacturer's instructions. cDNA was synthesised with 500 ng of extracted RNA in 20 µL using a High Capacity cDNA Reverse Transcription Kit (Applied Biosystems). qPCR was performed using Taqman Universal PCR Master Mix 2× (Thermo Scientific), with *C9orf72* Taqman probes (Thermo Scientific) and GAPDH Taqman probes (Thermo Scientific) for normalisation in an Applied Biosystems StepOne Plus system. *C9orf72* expression analysis was performed using the $\Delta\Delta$Ct method[1].

Transmission electron microscopy.

0.75 % phosphotungstic acid (PTA) stain solution was pH-adjusted to pH 7.4 with NaOH to prevent PSNA nanoparticle disassembly. A freshly glow discharged 300 mesh copper grid with a thin carbon film was applied to a 10 µl droplet of vesicle suspension (carbon side down) and incubated for 2 minutes. The grid was gently blotted, then incubated on a droplet of 0.75% phosphotungstic acid (pH 7.4) for 10 seconds, blotted, incubated on a droplet of MilliQ water for 10 seconds, blotted and air dried. Negative staining was accomplished by imaging the grids prior to the MilliQ wash. Grids were imaged using a JEOL 1400 TEM operated at 120kV and equipped with a Gatan Rio digital camera.

Quantification of oligo loading.

Known concentrations of Cy5.5-labelled oligo in PBS were used to build a standard curve with which to quantify fluorescence from PSNA particles measured in a BMG Labtech ClarioStar plate reader.

Supplementary Figures

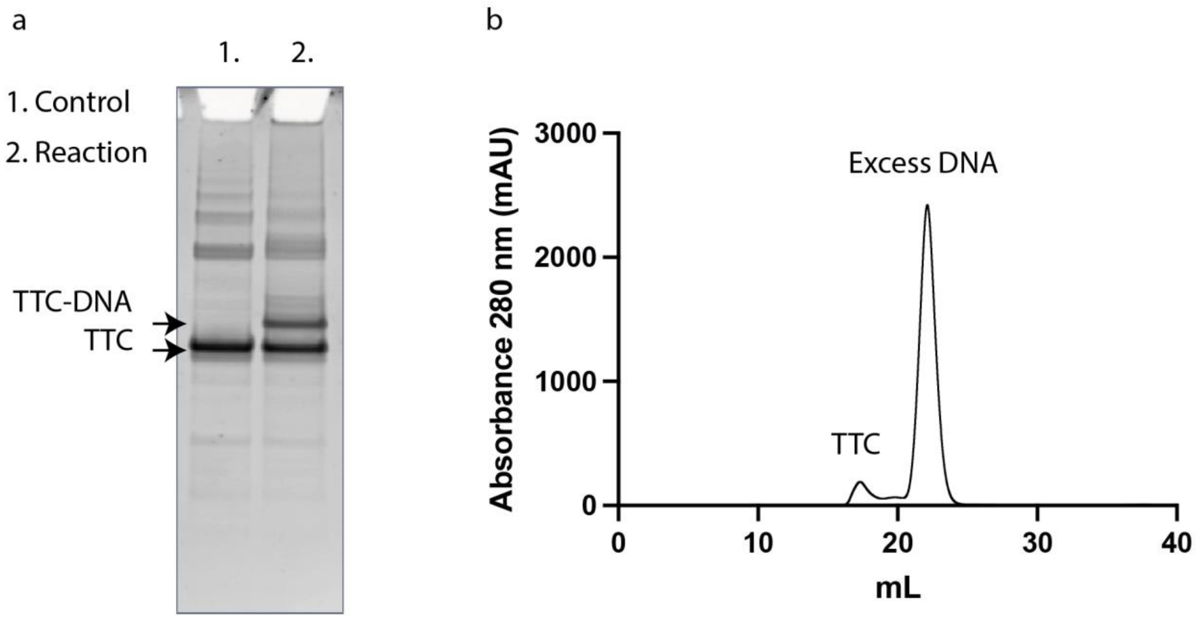

Supplementary information figure 1: Functionalization of TTC cysteines with a maleimide-DNA oligo. **a.** PAGE analysis. Lane 2 (reaction) contains an additional band with lower mobility than the control (Lane 1, unfunctionalized TTC) consistent with successful conjugation. Gel: Bolt 4-12 % polyacrylamide stained with Sypro Ruby. **b.** Size-exclusion purification of TTC-DNA from excess DNA using a Sephadex Peptide column. The first peak corresponds to the conjugated TTC, the second to unreacted, excess DNA. The TTC-DNA peak is further purified using an Amicon ultrafilter (30 kDa) to remove DNA and concentrate the sample.

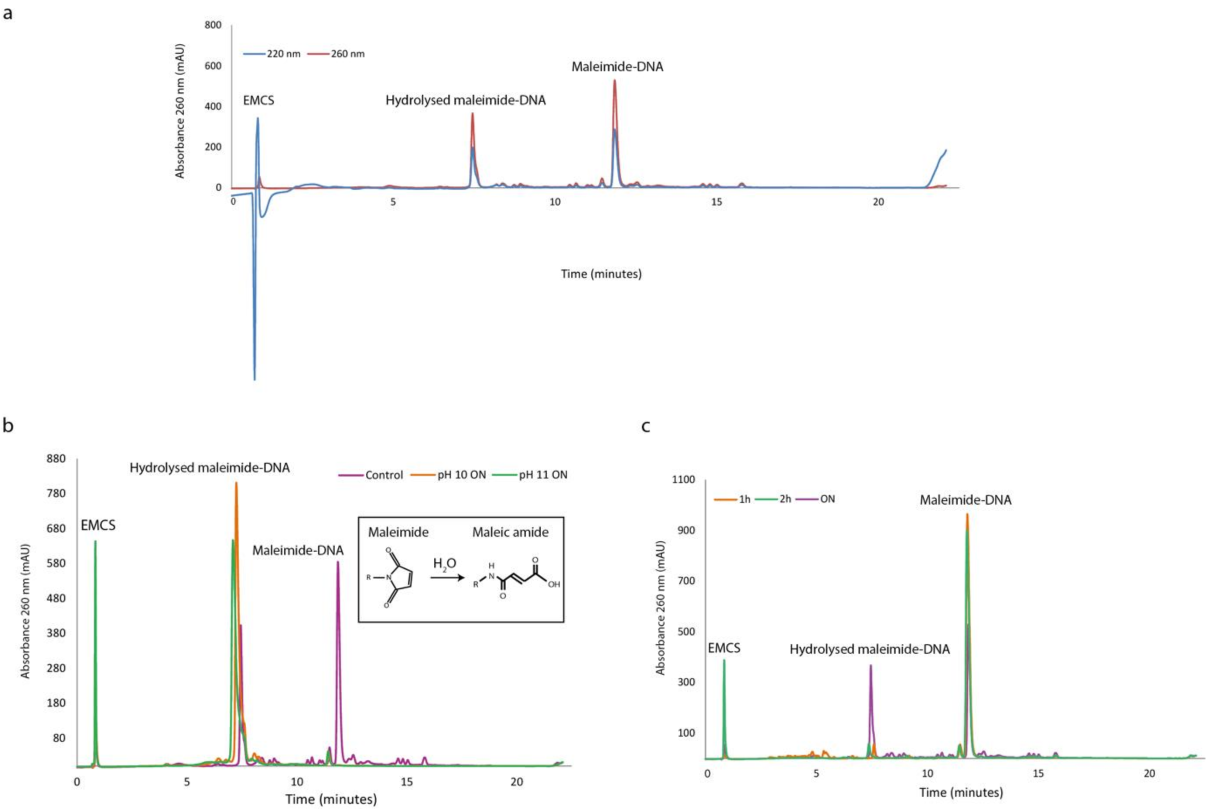

Supplementary information figure 2: Reaction of amine-modified DNA with hetero-bifunctional crosslinker EMCS to create maleimide-functionalized DNA. Each chromatogram corresponds to injection of 1 nmol of maleimide-DNA conjugate. **a.** HPLC chromatograms of reaction products at 260 nm (DNA) and 220 nm (DNA and polymer). **b.** Identification of the peaks shown in a. Maleimide-DNA conjugates were incubated overnight at pH 10 and 11 to hydrolyse the maleimide. The peak at 7.5 min increases as the peak at 13 min disappears. Since hydrolysis under basic conditions opens the maleimide ring to produce a maleic amide, we can conclude that the peak at 7.5 min is the hydrolysed maleimide and the peak at 13 min is the intended maleimide-DNA conjugate. **c.** Optimization of maleimide-DNA reaction time to minimise maleimide hydrolysis. 100 nmol reactions were incubated for 1 and 2 h, and 1 nmol of each condition was injected. The time point that minimises maleimide hydrolysis is 1 h.

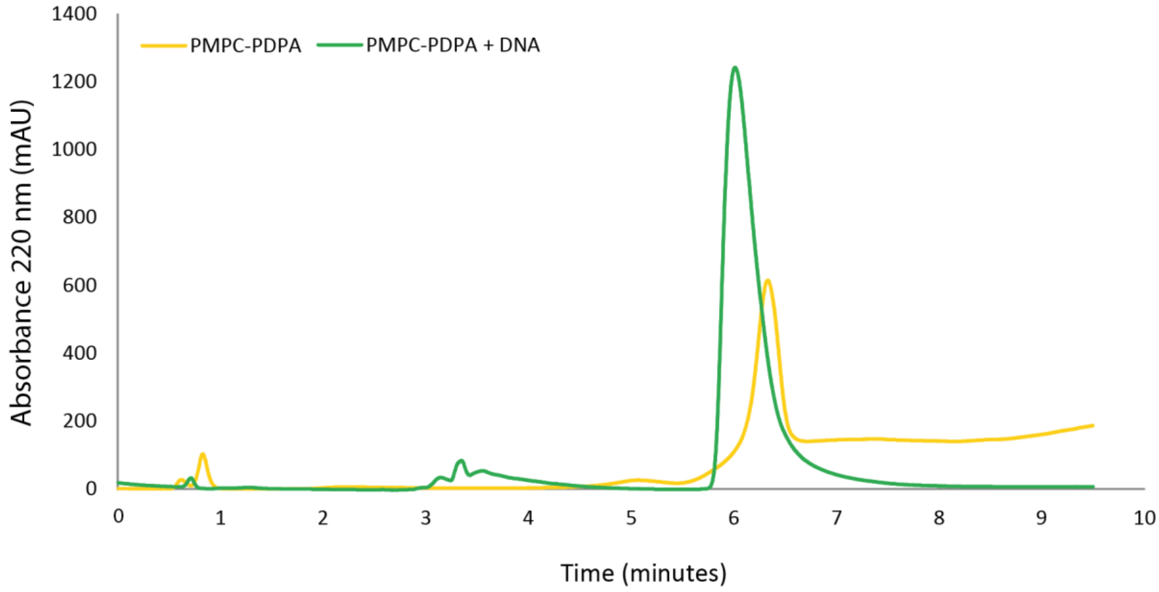

Supplementary information figure 3: Non-specific binding between charged $PMPC_{25}$-$PDPA_{72}$ and DNA oligonucleotides. Elution profile of $PMPC_{25}$-PDPA72 molecules in the presence and absence of a non-maleimide-functionalized DNA oligo. The chromatogram at 220 nm shows both DNA and polymer absorption. Each 50 µL sample comprised 800 µg of $PMPC_{25}$-$PDPA_{72}$ in 0.05% trifluoroacetic acid; DNA was at a concentration of 300 µM. The shift in the polymer elution peak when DNA is present is consistent with non-specific (electrostatic) interaction between species. HPLC flow rate 1 mL/min with elution program (minute, %B): (0, 0), (9, 100), (9.1, 0), (9.5, 0).

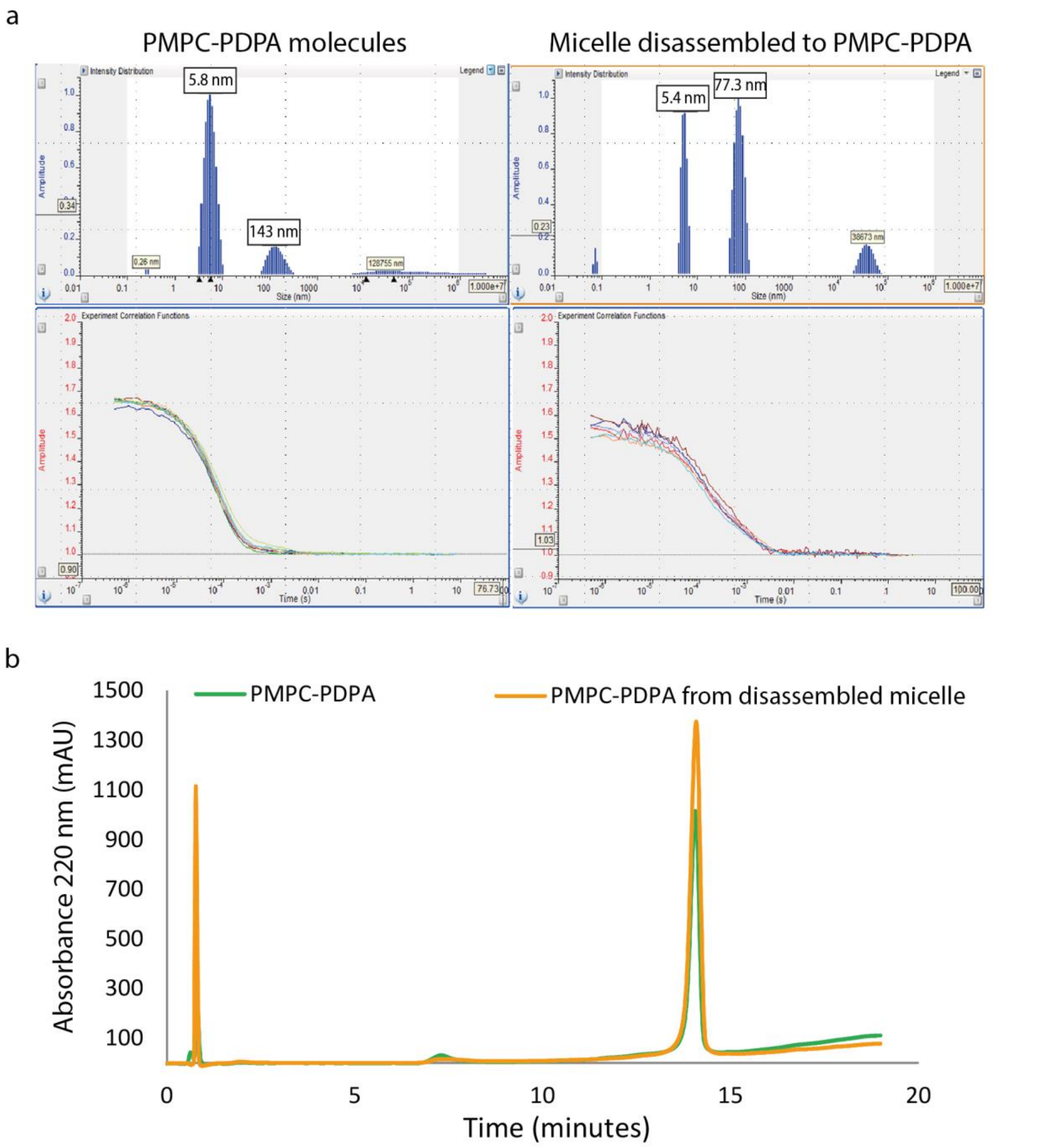

Supplementary information figure 4: Reversible micelle formation. **a.** Dynamic Light Scattering (DLS) analysis to compare $PMPC_{25}$-$PDPA_{72}$ polymer before and after a cycle of micelle formation (as described in the Experimental section) followed by micelle disassembly by acidification using TFA. 100 µL of $PMPC_{25}$-$PDPA_{72}$ polymers and disassembled micelles at 0.55 and 0.25 mg/mL respectively were loaded into the DLS cuvette. 8 measurements at 10 s each, whose correlation functions are shown, were used to produce the size distributions. Both samples show a dominant peak that corresponds to the hydrodynamic radius of the $PMPC_{25}$-$PDPA_{72}$ polymer in the range 5-6 nm. **b.** HPLC chromatogram of disassembled $PMPC_{25}$-$PDPA_{72}$ micelles. 50 µL of a 55 µL sample containing 0.55 mg of $PMPC_{25}$-$PDPA_{72}$ control or 1 mg reversed $PMPC_{25}$-$PDPA_{72}$ micelles were loaded into the column. The elution profile of the disassembled micelles is identical to that of the control, confirming the reversibility of micellar structure formation. HPLC flow rate 1 mL/min with elution program (minute, %B): (0, 5), (5, 5), (17, 80), (18.5, 80), (19,5).

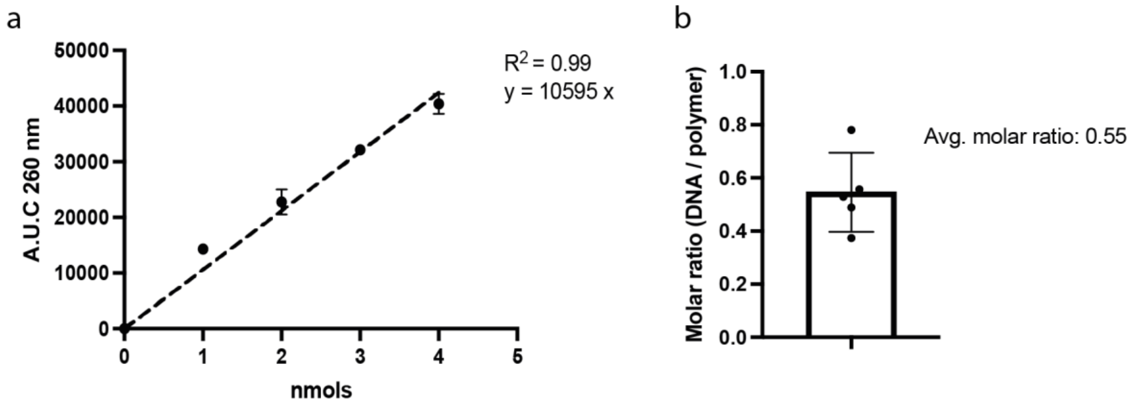

Supplementary information figure 5: Quantification of DNA conjugated to $PMPC_{25}$-$PDPA_{72}$ after ultrafiltration. **a.** A calibration curve constructed from chromatograms of known quantities of DNA is used to quantify the amount of DNA in DNA-polymer samples (A.U.C, area under the curve). Quantities of DNA were chosen to span the dynamic range of the HPLC detector. **b.** Corresponding estimates of the composition of the polymer-DNA conjugate used to prepare polymersomes. Data presented is mean and SD (n = 5).

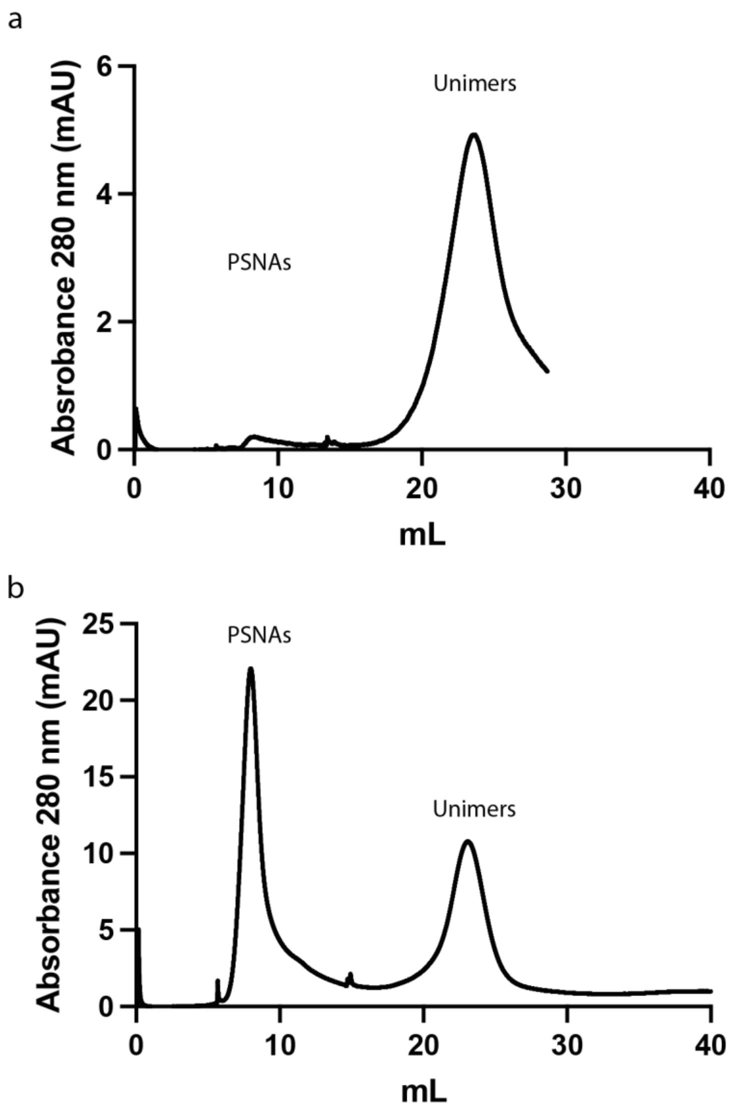

Supplementary information figure 6: a. Size Exclusion chromatograms of PSNA preparations. **a.** Failed PSNA assembly with 100% DNA-PMPC-PDPA conjugates. **b.** The corresponding chromatogram of PSNAs prepared using a 1:1 molar ratio of unfunctionalized and DNA-functionalized PMPC-PDPA. The peak at 8-12 mL corresponds to the expected PSNAs (DNA-functionalized polymersomes), which elute in the void volume.

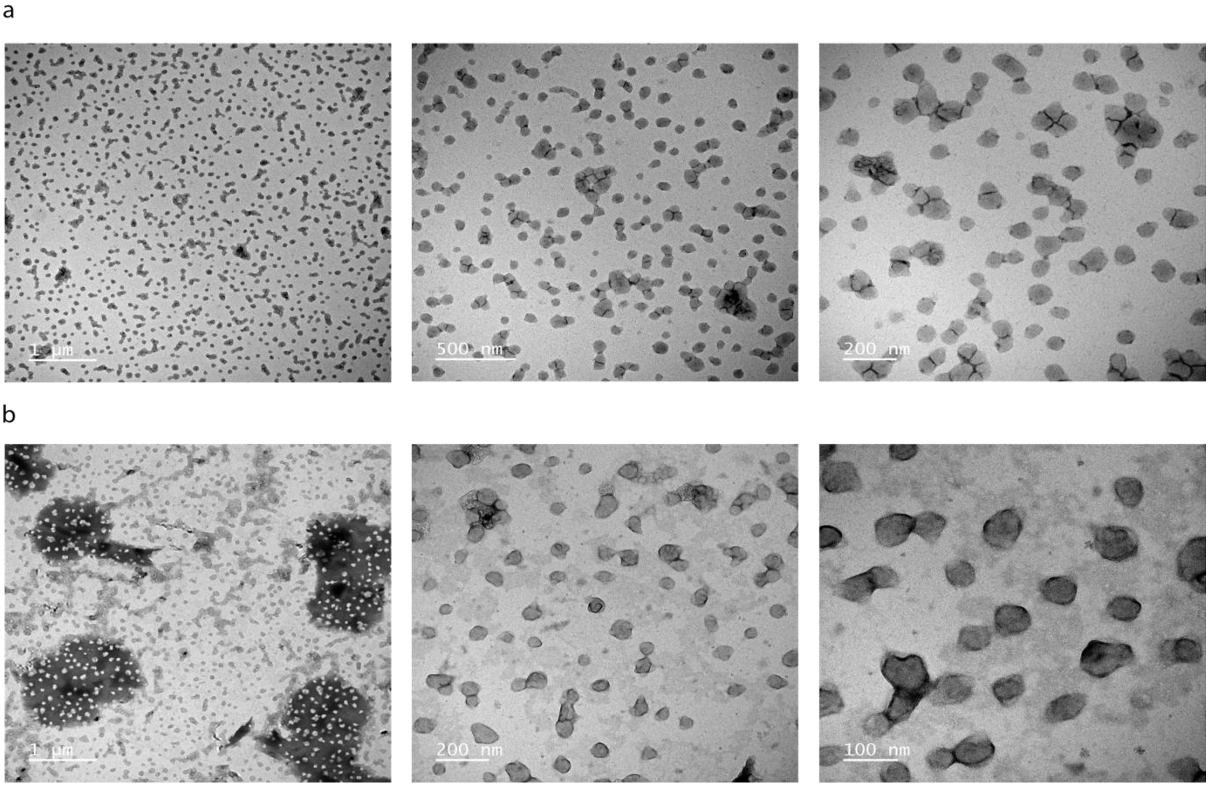

Supplementary information figure 7: Transmission Electron Micrographs of assembled PSNAs stained with pH-adjusted 0.75% Phosphotungstic Acid. **a.** Positively stained PSNA nanoparticles. **b.** Negatively stained PSNA nanoparticles. Image magnification increases from left to right. Scale bars, 1 μm, 200 nm, and 100 nm.

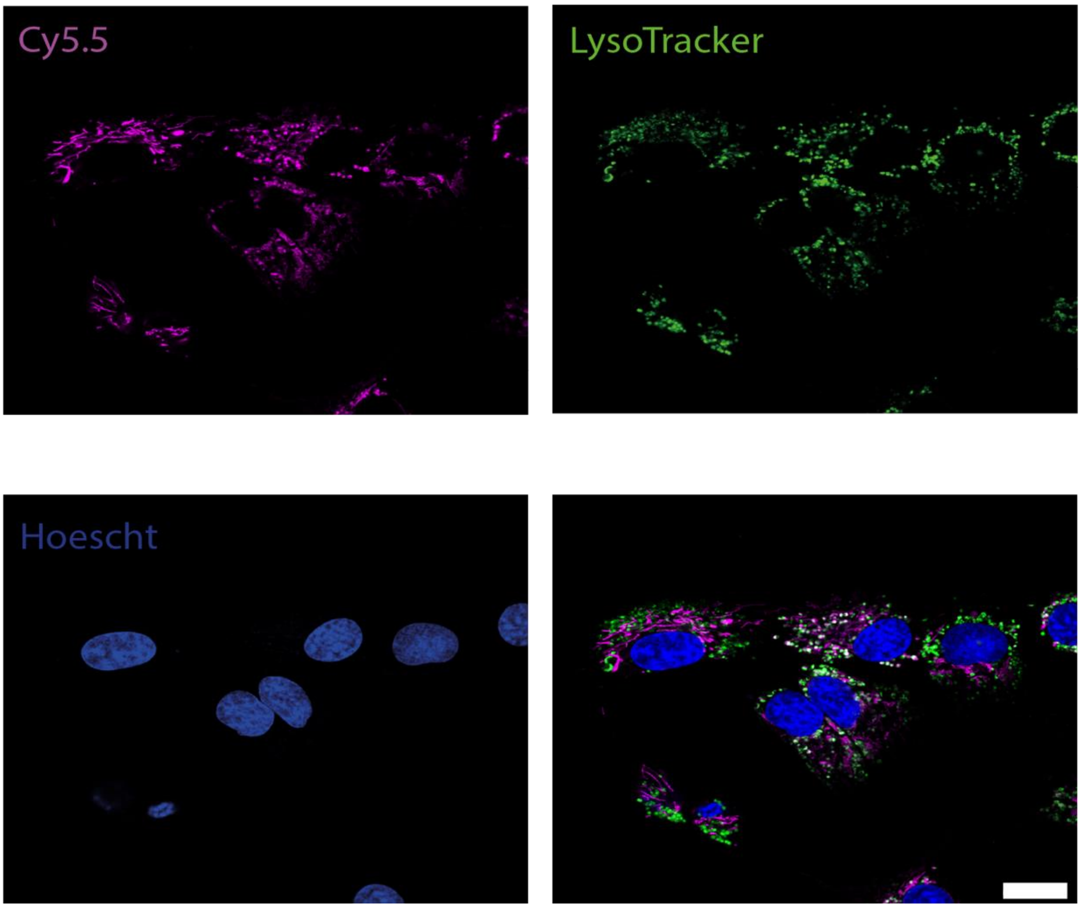

Supplementary information figure 8: Delivery of Cy5.5-labelled DNA oligos to human primary myoblasts. 30,000 cells were seeded and simultaneously treated overnight with 15,000 PSNAs per cell loaded with approximately 100 Cy5.5 oligos per particle. Cells were labelled with LysoTracker Green DND26 and Hoescht 33342 and imaged. Magenta – Cy5.5-labelled oligos; green - acidified lysosomal compartments; blue - cell nucleus. Objective 40×, scale bars 50 µm.

a

b

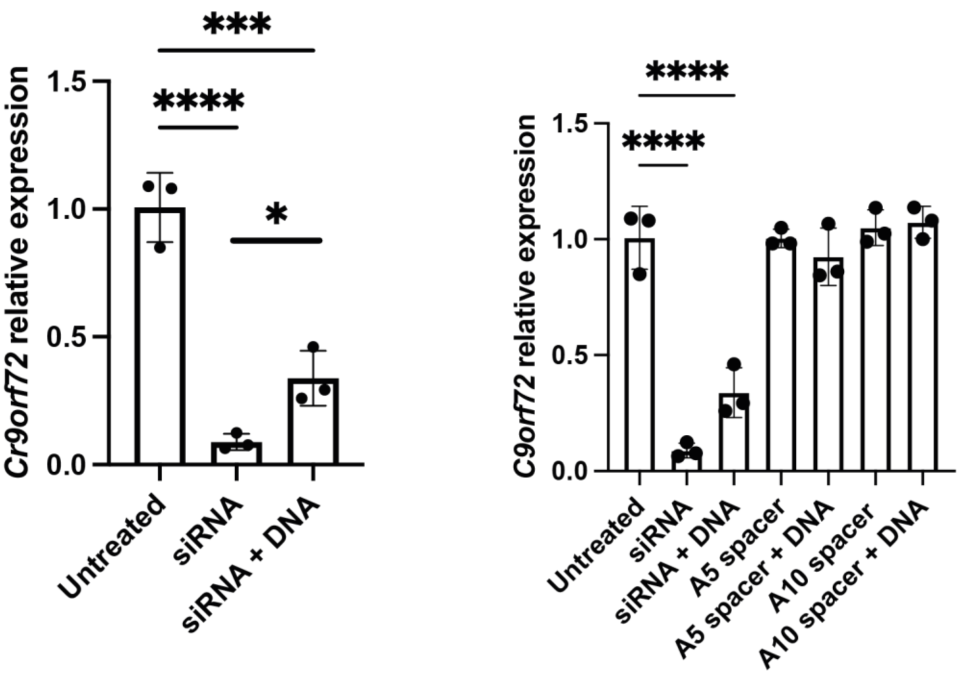

Supplementary information figure 9: a. Effect on knockdown efficiency of hybridizing a DNA oligo to a 3' extension of the sense strand of siRNA. 50,000 NSC34 cells were transfected with 50 nM siRNA (final concentration in the well) complexed with Lipofectamine™ RNAiMAX. There is a significant difference between the activities of siRNA with and without the DNA oligo. Data presented are mean and SD (n = 3). All samples were compared using a one-way ANOVA with Dunnett's multiple comparison test. *** $p < 0.001$, **** $p < 0.0001$. An unpaired two-tailed t-test was used to compare siRNA to siRNA + DNA. *$p < 0.05$. **b.** Effect of adding poly-adenosine spacers between the siRNA duplex and the duplex formed by hybridization of the 3' RNA extension to a complementary DNA oligo. When linkers with 5 or 10 adenosines (A_5, A_{10}) were added to the siRNA sense strand siRNA activity was abolished. Data presented are mean and SD (n = 3). Samples were compared using a one-way ANOVA with Dunnett's multiple comparison test **** $p < 0.0001$.

Supplementary Table: Oligonucleotide Sequences

Name	Sequence
Amino DNA for PSNA conjugation in FAM experiments	5' Amino-C6-GGGCAAACCTCGGCTTACCTGAAAT
3' FAM Ncomp DNA	GGGCAAACCTCGGCTTACCTGAAAT-3’ 6-FAM
3' FAM Comp DNA	ATTTCAGGTAAGCCGAGGTTTGCCC-3’ 6-FAM
Cy5.5 Comp DNA	ATTTCAGGTAAGCCGAGGTTTGCCC-3’ Cy5.5
Amino DNA for PSNA conjugation	5' Amino-C6-TGATCACTATGGTAAACTCTAAACT
Amino DNA for TTC functionalization	5'Amino-C6-AGTTTAGAGTTTACCATAG
PSNA DNA for siRNA experiments (supp. Fig. 8)	TGATCACTATGGTAAACTCTAAACT
C9orf72 siRNA sense	5'phos-CGAAAAGGAAGAAUAUGGAUGAGUUUAGAGUUUACCAU
C9orf72 siRNA antisense	5'phos-UCCAUAUUCUUCCUUUUCGAA
C9orf72 siRNA sense scramble	5'phos-UCUUCCUUACCUUUAUUGCUUAGUUUAGAGUUUACCAU
C9orf72 siRNA antisense scramble	5'phos-GCAAUAAAGGUAAGGAAGAUU
C9orf72 siRNA sense A5	5'phos-CGAAAAGGAAGAAUAUGGAUAAAAAAGUUUAGAGUUUACCAU
C9orf72 siRNA sense A10	5'phos-CGAAAAGGAAGAAUAUGGAUAAAAAAAAAAAGUUUAGAGUUUACCAU
All RNA strands were synthesized using ribonucleotides	

Supplementary References

1. K. J. Livak and T. D. Schmittgen, *Methods*, 2001, **25**, 402–408.
